# Doxorubicin induced ROS-dependent HIF1α activation mediates blockage of IGF1R survival signaling by IGFBP3 promotes cardiac apoptosis

**DOI:** 10.18632/aging.204466

**Published:** 2023-01-03

**Authors:** Su-Ying Wen, Ayaz Ali, I-Chieh Huang, Jian-Sheng Liu, Po-Yuan Chen, Vijaya Padma Viswanadha, Chih-Yang Huang, Wei-Wen Kuo

**Affiliations:** 1 Department of Dermatology, Taipei City Hospital, Renai Branch, Taipei 11260, Taiwan; 2 Department of Cosmetic Applications and Management, Mackay Junior College of Medicine, Nursing and Management, Taipei 112, Taiwan; 3 Department of Health Care Management, National Taipei University of Nursing and Health Sciences, Taipei, Taiwan; 4 Department of Biological Science and Technology, China Medical University, Taichung 404, Taiwan; 5 China Medical University Beigang Hospital Thoracic Department, Yunlin 651, Taiwan; 6 Department of Biotechnology, Bharathiar University, Coimbatore 641046, India; 7 Center of General Education, Buddhist Tzu Chi Medical Foundation, Tzu Chi University of Science and Technology, Hualien 970, Taiwan; 8 Department of Medical Research, China Medical University Hospital, China Medical University, Taichung 404, Taiwan; 9 Department of Medical Laboratory Science and Biotechnology, Asia University, Taichung 413, Taiwan; 10 Graduate Institute of Biomedical Sciences, China Medical University, Taichung 404, Taiwan; 11 Cardiovascular and Mitochondrial Related Disease Research Center, Hualien Tzu Chi Hospital, Buddhist Tzu Chi Medical Foundation, Hualien 970, Taiwan; 12 Ph.D. Program for Biotechnology Industry, China Medical University, Taichung 406, Taiwan

**Keywords:** doxorubicin, reactive oxygen species, cardiomyocyte apoptosis, hypoxia-inducible factor 1α, IGF-binding protein-3

## Abstract

Doxorubicin (Dox) causes the generation of intracellular reactive oxygen species (ROS) and inactivates insulin-like growth factor 1 (IGF1) signaling, leading to cardiomyocyte apoptosis. IGF-binding protein 3 (IGFBP3) is the most abundant circulating IGF1 carrier protein with high affinity, which has been reported to mediate ROS-induced apoptosis. Hypoxia-inducible factor 1α (HIF1A), an upstream protein of IGFBP3 is regulated by prolyl hydroxylase domain (PHD) through hydroxylation. In this study, we investigated the role of IGFBP3, HIF1A, and PHD in Dox-induced cardiac apoptosis.Cells challenged with 1 μM Dox for 24 h increased ROS generation, augmented intracellular and secreted IGFBP3 levels, and reduced IGF1 signaling. Further, we showed that Dox enhanced the extracellular association of IGF1 with IGFBP3. Moreover, echocardiography parameters, especially ejection fraction (EF) and fractional shortening (FS) were significantly reduced in ventricle tissue of Dox challenged rats. Notably, siRNA approach against IGFBP3 or an anti-IGFBP3 antibody rescued Dox-induced cardiac apoptosis, mitochondrial ROS, and the decrease in the IGF1 signaling activity. Furthermore, silencing HIF1A either using siRNA or inhibitor downregulated intracellular IGFBP3, rescued apoptosis, mitochondrial generation, and reduction in IGF1 signaling. Finally, western blot data revealed that ROS scavenger reversed Dox-induced cardiac apoptosis, increased levels of HIF1A and secreted IGFBP3, and decreased IGF1 survival signaling and PHD expression.These findings suggest that Dox-induced ROS generation suppressed PHD, which might stabilize nuclear HIF1A protein, leading to increased IGFBP3 expression and secretion. This in turn results in enhanced extracellular association of the latter with IGF1 and blocks IGF1 pro-survival signaling and may result in inducing cardiac apoptosis.

## INTRODUCTION

Doxorubicin (Dox), a potent antibiotic and chemotherapeutic anthracycline drug, is commonly used for the treatment of a wide range of cancers. However, it possesses significant cardiotoxicity, and causes degenerative cardiomyopathy and progressive myocardial damage for long after the cessation of treatment. It results in a range of symptoms, including subclinical myocardial dysfunction to severe heart failure and even death [1–3]. Previous studies indicated that cardiac damage following Dox treatment is induced through the generation of reactive oxygen species (ROS) [4] and intracellular ROS-dependent cardiac apoptosis, and is mediated by the activation of JNK, p38, and p53 [5].

Insulin-like growth factor binding protein 3 (IGFBP3), isolated for the first time from human plasma in 1986 [6] is predominantly synthesized in the liver and acts as an autocrine/paracrine regulator of insulin-like growth factor I (IGF1) in the brain, bone, muscle, kidney, and heart tissues [7–9]. IGFBP3 shares a three-domain structure with the other five high-affinity IGFBPs [10]. The conserved N-terminal domain containing 12 cysteine residues is the primary site for IGF binding. The mid-region separates the N-terminal domain from the C-terminal domain. Intriguingly, the C-terminal domain also contains residues involved in the interaction with IGF [11–13]. Furthermore, IGFBP3 exerts anti-proliferative effects in many cells by blocking the ability of IGF1 to activate the IGF1 receptor (IGF1R) [14]. Based on this evidence, we hypothesized that exposure of myocardial cells to Dox may enhance IGFBP3 expression and secretion via autocrine or paracrine routes and thereby induce cardiomyocyte apoptosis.

Previous studies demonstrated that Dox induces cardiotoxicity through ROS-mediated signaling and inhibition of the IGF1-PI3K-AKT survival pathway, thereby promoting cell apoptosis [15]. Mitochondria are likely the main source of ROS in cells [16]. Furthermore, previous studies indicated that mitochondria-derived ROS can inhibit the activity of prolyl hydroxylase domain (PHD) proteins [17]. Three PHD-containing paralogs have been identified: (PHD1, PHD2, and PHD3) [18, 19], of which, PHD3 expression is upregulated in a diseased heart [20]. Under normoxic conditions, hypoxia induced factor (HIF) is hydroxylated by the PHD-containing enzymes and also undergoes polyubiquitination and proteasomal degradation by the von Hippel-Lindau protein [21]. In order to sense oxygen, PHD-containing proteins respond to a variety of other signals, including changes in NO and ROS concentrations [22]. The mechanisms underlying the regulation of cardiomyocyte apoptosis by HIF are unclear, but they may involve transcriptional activation of genes such as IGFBP3, whose protein product inhibits the pro-survival signaling pathway. In addition, it is unclear whether PHD-HIF1A signaling may affect IGFBP3 expression and is relevant for Dox-induced apoptosis.

In the present study, we have verified whether the molecular mechanisms of Dox-induced cell death involve downregulation of cardiac IGF1 pro-survival signaling through the increased association of IGF1 with IGFBP3 secretion and subsequent sequestration. Additionally, we explored whether ROS-regulated PHD proteins mediated the intracellular activation of HIF1A-IGFBP3 signaling to increase IGFBP3 secretion in relevance to the Dox-induced cardiac apoptosis. In particular, we utilized Dox-treated rat cardiac cells (the H9c2 cell line) and heart ventricle samples from rats administered with Dox. Our study revealed the regulatory role of Dox-induced proteins, in particular IGFBP3 and components of the HIF1A-IGF1 pathway in cardiac apoptosis, which may be important for the adjustment or replacement of cancer therapies that involve accumulation of excessive Dox levels.

## RESULTS

### Dox-induced ROS production and apoptosis in a dose-dependent manner in embryo-derived cardiac cells

**Figure 1:**
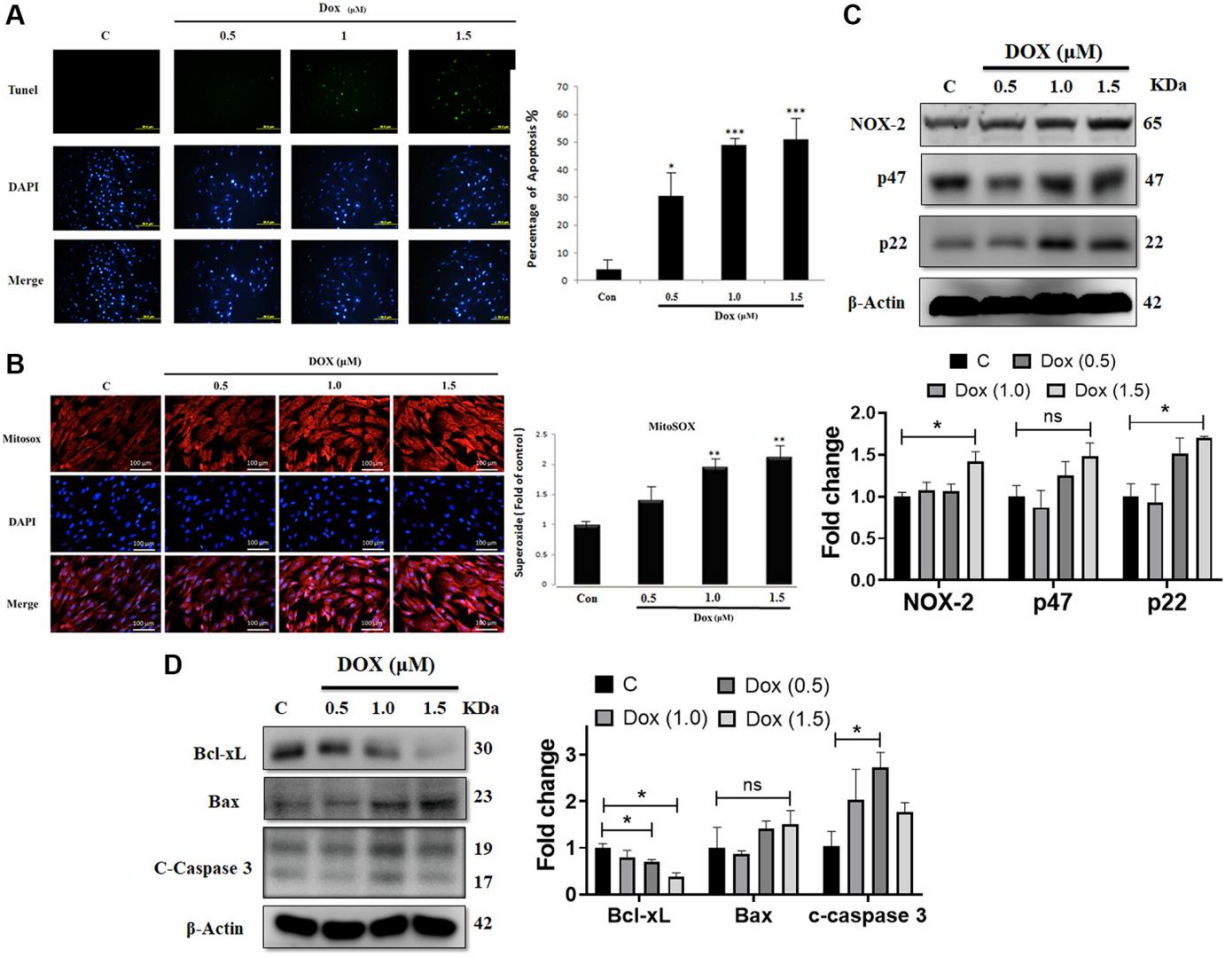
**Doxorubicin increases generation of ROS and augments apoptosis in cardiac cells.** H9c2 cells challenged with increasing doses of doxorubicin (Dox) for 24 h were harvested. (**A**) Apoptosis as detected using the TUNEL assay. (**B**) Mitochondrial superoxide production measured using MitoSOX staining. (**C**) The protein expression levels of the NADPH oxidase subunits NOX-2, p47, and p22 were detected using immunoblotting. (**D**) Protein levels of Bcl-xL, Bax, and cleaved caspase 3 were analyzed using western blot. Data are presented as mean ± standard deviation (*n* = 3). Scale bar represents 100 μm. Statistical significance is indicated as follows: ^*^P < 0.05, ^**^P < 0.01, ^***^P < 0.001.

Earlier studies indicated that Dox can induces oxidative stress and apoptotic cell death. To evaluate the impact of Dox on embryo-derived cardiac cells (the H9c2 cell line), we used the TUNEL assay. Treatment with Dox at 0.5, 1.0, or 1.5 μM for 24 h induced cell apoptosis in a dose-dependent manner in H9c2 cells (Figure 1A). Further, MitoSOX staining was performed to evaluate the effect of Dox challenge on mitochondrial superoxide generation. Mitochondrial superoxide generation was significantly increased by Dox administration in a dose-dependent manner (Figure 1B). Moreover, we analyzed the effect of Dox on various oxidative stress markers. Western blot analysis indicated that the protein expression levels of pro-oxidative stress markers, such as the NADPH oxidase subunits NOX2, p47, and p22^phox^ were elevated following Dox challenge (Figure 1C). Although there was a dose-dependently increased trend, however, only 1.5 μM reached to significant level. Additionally, we evaluated the impact of Dox on different apoptosis markers. Immunoblotting data showed that the protein expression level of the anti-apoptotic marker (Bcl-xL) was downregulated significantly, whereas that of the pro-apoptotic markers (Bax and caspase 3) were elevated by Dox challenge (Figure 1D), although there is no significant difference in the levels of Bax compared with control, while in the case of caspase 3, only 1 μM Dox dose had a significant difference. These results confirm that Dox can induce ROS generation and apoptosis mediated cell death in embryo-derived cardiac cells. Furthermore, following a 6-week Dox treatment, rat cardiac functions were evaluated using an echocardiogram (Table 1). We observed that several parameters, especially ejection fraction (EF) and fractional shortening (FS), that assess the function of the left ventricle, were significantly lower in Dox-treated rats. This is in line with the notion that Dox induces cardiac damage.

**Table 1:** Heart size and cardiac functions in rats.

	**WT**	**Dox treated**
	**(*n* = 5)**	**(*n* = 5)**
BW (g)	255 ± 3.83	248 ± 6.32
IVSd (mm)	1.22 ± 0.09	1.01 ± 1.01^**^
LVIDd (mm)	8.00 ± 0.59	7.05 ± 0.5^*^
LVPWd (mm)	1.19 ± 0.20	0.84 ± 0.06^*^
IVSs (mm)	2.34 ± 0.08	1.92 ± 0.35
LVIDs (mm)	5.31 ± 0.81	4.32 ±0.50
LVPWs (mm)	2.24 ± 0.09	1.45 ± 0.42^*^
EDV (Teich) (ml)	1.20 ± 0.28	0.97 ± 0.08
ESV (Teich) (ml)	0.37 ± 0.16	0.21 ± 0.06
EF (Teich) (ml)	76.85 ± 2.36	59.54 ± 0.89^***^
% FS	40.70 ± 2.07	27.86 ± 0.45^***^
SV (Teich) (ml)	0.86 ± 0.11	0.69 ± 0.09^**^
LVd Mass (ASE) (g)	1.12 ± 0.01	0.97 ± 0.02^**^
LVS Mass (ASE) (g)	1.20 ± 0.13	1.05 ± 0.01
CO, L/min	197.8 ± 2.43	131.1 ± 3.22^***^

Abbreviations: BW: body weight; IVSd: end-diastolic interventricular septal thickness; LVIDd: left ventricular internal dimension end (diastole); LVPWd: left ventricular posterior wall thickness; IVSs: interventricular septum thickness at end (systole); LVIDs: left ventricular internal dimension at end (systole); LVPWs: left ventricular posterior wall thickness at end (systole); EDV: end-diastolic volume; ESV: end-systolic volume; EF: ejection fraction; FS: fractional shortening; SV: stroke volume; LVd mass: left ventricular cavity diameter; LVS Mass: left ventricular mass index; CO: cardiac output. Data are presented as the mean ± standard error of the mean. Statistical significance of differences is indicated as follows: ^*^P < 0.05, ^**^P < 0.01, ^***^P < 0.001 in comparison with the control group.

### Dox increases cardiac IGFBP3 expression and secretion as well as decreases cardiomyocyte survival *in vitro* and *in vivo*

**Figure 2:**
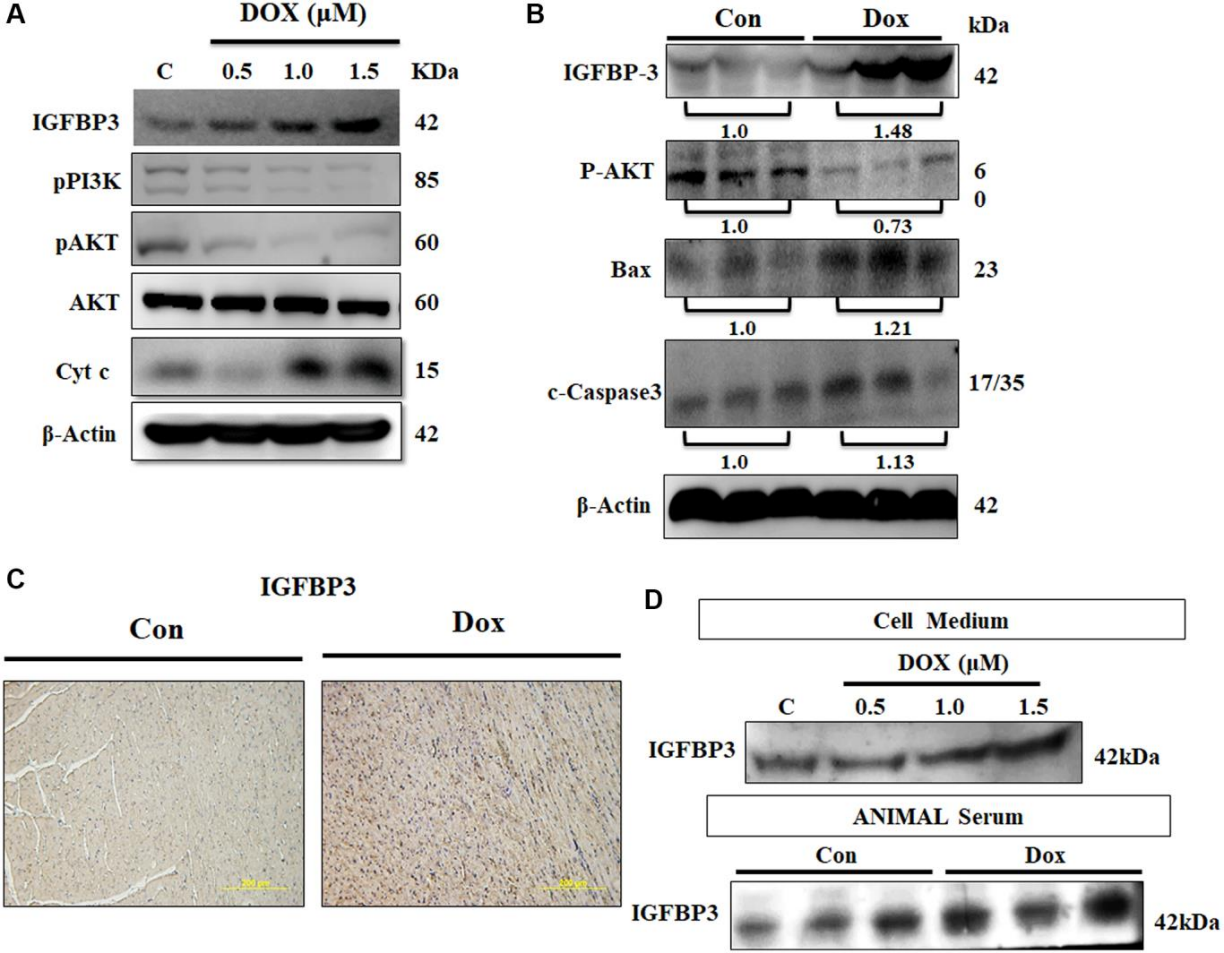
**Doxorubicin increases expression and secretion of IGFBP3, and decreases cardiac cell survival *in vitro* and *in vivo*.** (**A**) H9c2 cells were incubated with Dox for 24 h at the indicated doses and protein expression of IGFBP3 and that of the components of survival signaling pathway were measured using immunoblotting. (**B**) Left ventricles of control and Dox-administered rats were isolated and the levels of IGFBP3 and survival-related proteins were analyzed using western blotting. (**C**) IGFBP3 expression from the rat left ventricle tissue was detected using immunohistochemistry. (**D**) H9c2 cells were challenged with Dox for 24 h at indicated doses and the amount of secreted IGFBP3 in both cell medium and in the animal serum of Dox-treated rats was examined using western blotting. The quantitative plot of IGFBP3 from the sera of doxorubicin challenged rats is included. Scale bar indicates 200 μm.

Expression of IGFBP3 and that of the components of IGF1 survival signaling pathway were assessed in cardiac cells exposed to 0.5, 1.0, and 1.5 μM Dox for 24 h. We found that IGFBP3 and cytochrome c protein levels were increased by Dox, whereas the protein expression levels of the survival pathway components, such as phosphorylated PI3K and AKT, were lower in the cells challenged with Dox (Figure 2A). Next, we measured the levels of these proteins in the ventricle heart tissue of Dox-treated and control rats using western blot and found similar changes to those seen in embryo-derived cardiac cells. Further, pro-apoptosis-related protein cleaved caspase 3 and Bax levels were highly increased in the ventricle tissue of Dox-administered rats (Figure 2B). Importantly, immunohistochemistry results showed that cardiac IGFBP3 accumulation was higher in the Dox-treated rats compared with that in the control animals (Figure 2C). Additionally, Dox treatment increased IGFBP3 secretion was revealed by higher IGFBP3 levels in both the cell culture medium of Dox-treated cardiac cells and serum of Dox-treated rats (Figure 2D). These results indicate that Dox could trigger cell apoptosis by increasing cardiac IGFBP3 expression and secretion coupled with the downregulation of IGF1 signaling.

### Downregulation of IGFBP3 prevents Dox-induced loss in IGF1 survival signaling, and increase in apoptosis and ROS generation

**Figure 3:**
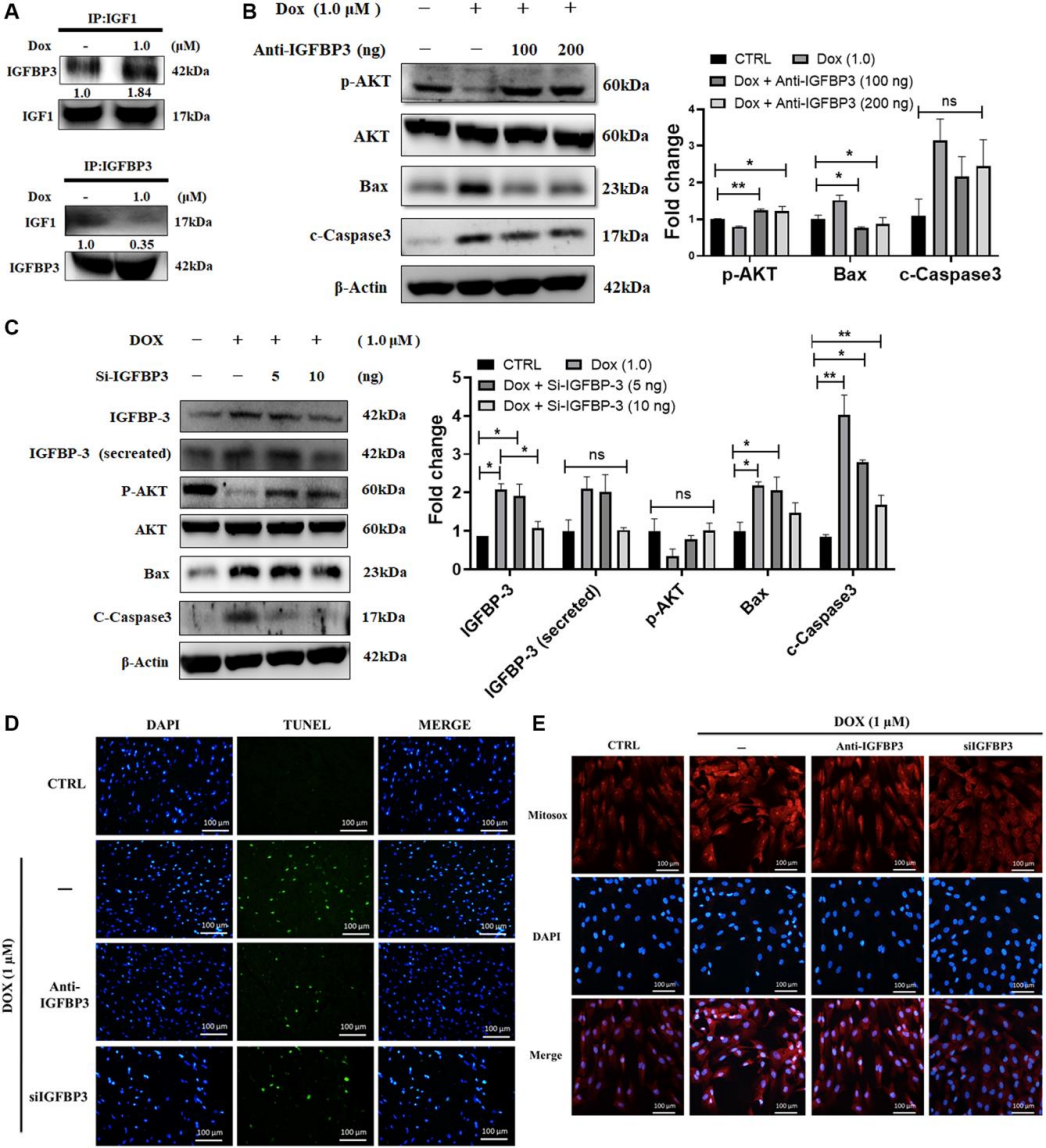
**Doxorubicin enhances extracellular binding of IGFBP3 to IGF1, blocking IGFI survival signaling and promoting cell apoptosis.** H9c2 cells were treated with 1.0 μM Doxorubicin (Dox) for 24 h. (**A**) Extracellular association of IGF1 with IGFBP3 was detected using co-IP. (**B**, **C**) Dox-challenged cells were incubated with either (**B**) anti-IGFBP3 antibody at indicated amounts or transfected with (**C**) *Igfbp3* siRNA. The levels of pro-survival and apoptosis-related proteins were analyzed using western blotting. (**D**, **E**) H9c2 cardiomyoblasts challenged with Dox (1.0 μM) for 24 h were either incubated with anti-IGFBP3 antibody and/or transfected with Si-*Igfbp3* and thereafter probed with TUNEL and MitoSOX reagents to evaluate the apoptotic cell death (**D**) and mitochondrial superoxide generation (**E**). Data are expressed as mean ± standard deviation (*n* = 3). Scale bar represents 100 μm. Statistical significance is showed as follows: ^*^P < 0.05, ^**^P < 0.01.

The above obtained data showed enhanced IGFBP3 expression and secretion following Dox treatment, which suppressed IGF1 signaling. Therefore, we hypothesized that IGFBP3, secreted in higher amounts into the extracellular space following exposure to Dox, binds IGF1 and blocks the survival pathway, thereby promoting cell apoptosis. Indeed, the co-IP data showed higher association of IGFBP3 with IGF1 in Dox-exposed cardiac cells (Figure 3A). Further, the levels of the phosphorylated IGF1 signaling pathway component phosphorylated AKT, were upregulated by the application of an anti-IGFBP3 antibody. Likewise, levels of the pro-apoptotic protein Bax and caspase 3, which were increased by Dox, were dose-dependently reduced by the treatment with an anti-IGFBP3 antibody (Figure 3B). Moreover, we applied *Igfbp3* siRNA approach to verify that Dox downregulated IGF1 survival signaling and increased pro-apoptotic protein levels by potentiating IGFBP3 expression. Treatment with Si-*Igfbp3* dose-dependently rescued the reduction of IGF1 survival signaling and increase in the levels of pro-apoptotic proteins (Figure 3C). Notably, TUNEL assay further revealed that Dox induced apoptosis (Figure 3D). Moreover, MitoSOX staining indicated that ROS generation was reverted to normal upon IGFBP3 inhibition using either anti-IGFBP3 or knockdown approach (Figure 3D, 3E). These results indicate that the Dox-induced inhibition of IGF1 survival signaling, cardiac cell apoptosis, and oxidative stress are possibly mediated by the enhancement of cardiac IGFBP3 expression.

### Effects of Dox on ROS generation, IGFBP3 and mediated cardiac apoptosis are mediated by HIF1A

**Figure 4:**
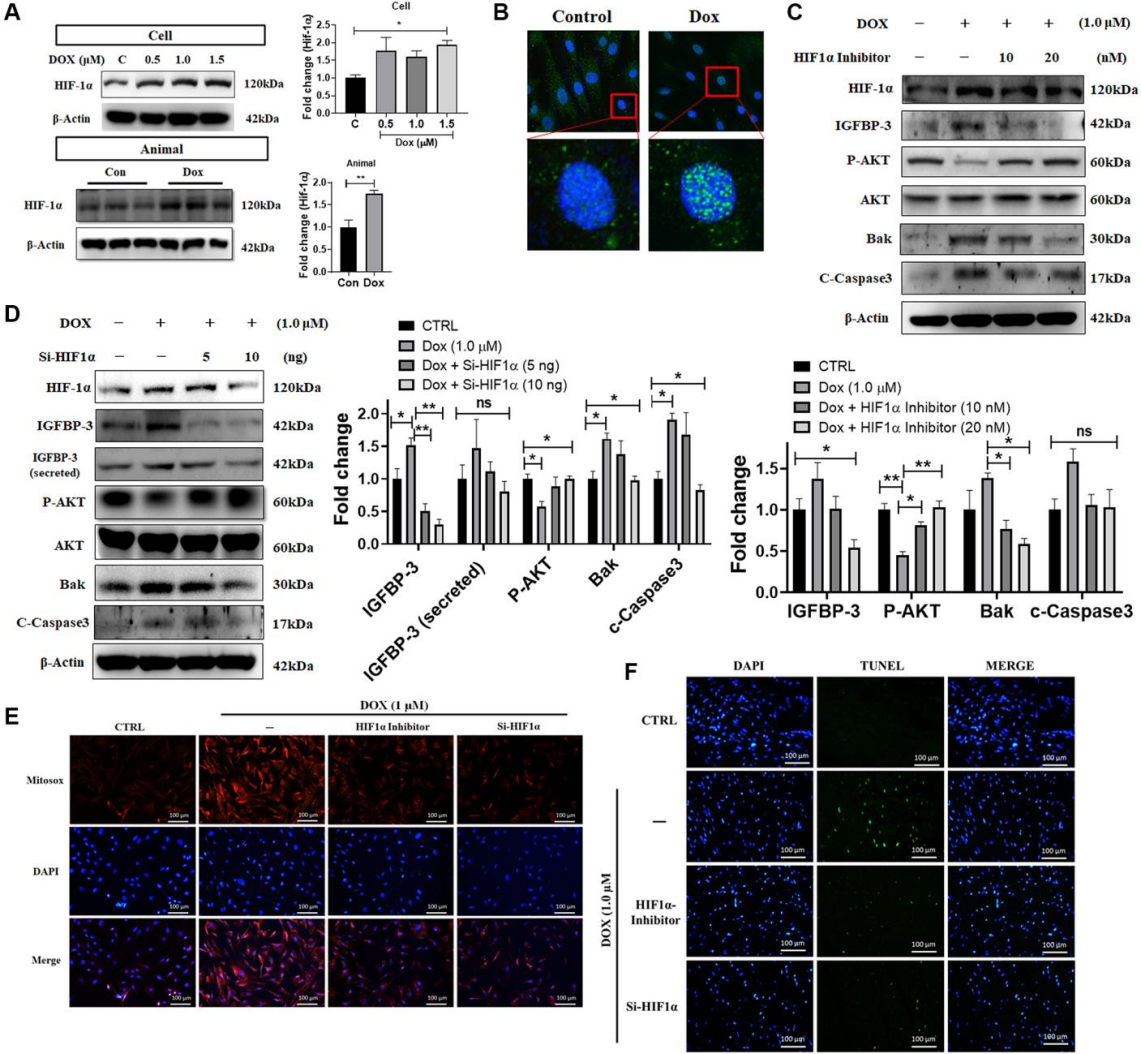
**Dox-induced ROS production, cell apoptosis, downregulation of pro-survival signaling, and upregulation of IGFBP3 expression are dependent upon HIF1A activity.** (**A**) H9c2 cells were incubated with increasing dose of Dox for 24 h. HIF1A protein levels were analyzed in H9c2 cells and animal cardiac tissue using western blotting. (**B**) Nuclear translocation of HIF1A in cells challenged with or without Dox were detected using fluorescence microscopy. Blue represents nucleus; green represents HIF1A. (**C**, **D**) Dox-exposed cells were treated with HIF1A inhibitor (**C**) or transfected with *Hif1a* siRNA (**D**). The levels of IGFBP3, pro-survival, and pro-apoptotic proteins in cells and cell culture medium (for secreted IGFBP3) were detected using western blotting. (**E**, **F**) Dox challenged H9c2 cells either treated with HIF1A inhibitor or transfected with si*Hif1a,* were incubated with TUNEL and MitoSOX reagents to assess apoptosis mediated cell death (**E**) and mitochondrial ROS production (**F**). Statistical significance difference was shown as ^*^P < 0.05, ^**^P < 0.01 from at least three independent experiments.

Previous studies have indicated that upon hypoxia exposure, cardiac HIF1A translocates from cytosol to the nucleus enhancing IGFBP3 expression and triggering cell apoptosis [14]. Therefore, we sought to verify whether HIF1A expression is induced by Dox. From the western blot analysis, we found that HIF1A protein expression was increased dose-dependently in H9c2 cardiac cells upon Dox challenge. Additionally, higher HIF1A levels were noted in the ventricles of Dox-treated rats compared with that in the normal group (Figure 4A). The observation of immunofluorescence microscopy demonstrated increment in Dox for 24 h induced HIF1A translocation from the cytosol to the nucleus (Figure 4B). Further, we examined the effects of *Hif1a* siRNA and HIF1A inhibitor on Dox-exposed cardiac cells. We found that Dox mediated decrease in the level of the pro-survival protein, phosphorylated AKT, was reverted using si*Hif1a* and HIF1A inhibitor in a dose-dependent manner. In addition, exposure to the HIF1A inhibitor also reversed Dox-induced upregulation of IGFBP3 expression and secretion (Figure 4C, 4D). Moreover, TUNEL and MitoSOX staining revealed that Dox-induced cardiac apoptosis and the enhanced mitochondrial ROS generation were reversed upon *Hif1a* knockdown and inhibition (Figure 4E, 4F). These results indicate that Dox-induced ROS production enhanced IGFBP3 expression and secretion, downregulated IGF1 survival signaling, and induced apoptosis of embryo derived cardiac cells in a HIF1A dependent manner.

### Dox-induced ROS inhibits PHD, thereby enhancing HIF1A-IGFBP3 signaling, leading to increased cardiac apoptosis

**Figure 5:**
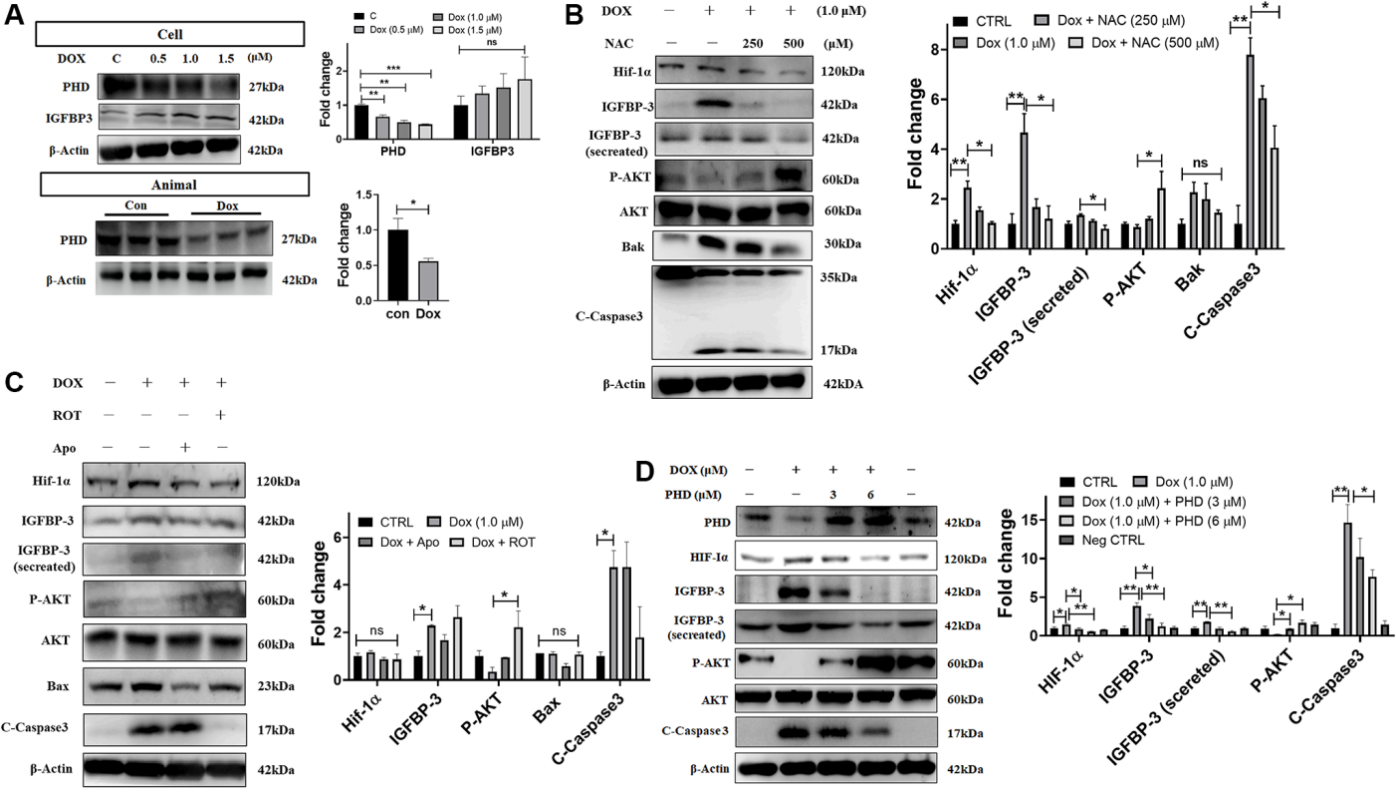
**Dox-induced mitochondrial ROS stabilizes HIF1A through the downregulation of PHD and promotes IGFBP3-induced cardiac apoptosis.** (**A**) H9c2 cells and tissues from rats administered with indicated concentrations of Dox were subjected to western blotting. (**B**, **C**) Dox-exposed cells were treated with the ROS scavenger NAC (**B**), mitochondria complex I inhibitor rotenone (Rot) or the NADPH oxidase inhibitor Apo (**C**). The harvested cellular extract was analyzed using western blotting. (**D**) Cells were transfected with a PHD overexpression plasmid of the indicated amount and levels of HIF1α, IGFBP3, pro-survival, and pro-apoptotic proteins in cells and cell culture medium (for secreted IGFBP3) were measured by western blot analysis. Data represents as the mean ± standard deviation of the mean (*n* = 3). Statistical significance is represented as follows: ^*^P < 0.05, ^**^P < 0.01.

Previous studies show that mitochondrial ROS suppress the expression of PHD proteins. Under normoxic conditions, HIF is hydroxylated by PHD-containing enzymes and undergoes polyubiquitination and proteasomal degradation by VHL E3 ubiquitin ligase [23]. Using western blotting, we measured the levels of the PHD protein in Dox challenged cardiac cells and tissue of rats administered with Dox and found that PHD expression was decreased in both models (Figure 5A). Further, we showed that the ROS scavenger NAC dose-dependently reversed the decrease in the levels of the phosphorylated pro-survival protein AKT, as well as attenuated the increase in the levels of HIF1A, IGFBP3 (cellular expression and secreted amount), and pro-apoptotic proteins Bak and caspase 3 induced by Dox exposure (Figure 5B). In order to determine the source of ROS following the exposure to Dox, we examined the effects of apocynin, an NADPH oxidase inhibitor that inhibits the cytosolic ROS, and rotenone, a complex 1 inhibitor that inhibits the generation of the mitochondrial ROS. We found that both inhibitors reversed the Dox-induced downregulation of PHD and p-PI3K levels and the upregulation of HIF1A, IGFBP3 (cellular expression and secreted amount), Bax, and pro-apoptotic caspase 3 (Figure 5C). Furthermore, we used PHD overexpression plasmid to investigate whether PHD downregulation is involved in the activation of HIF1A by Dox. Immunoblotting data showed that overexpression of PHD reversed the Dox-induced enhancement of the levels of HIF1A, IGFBP3 (cellular expression and secreted amount), and pro-apoptotic proteins as well as the attenuation of p-AKT levels (Figure 5D). These results indicate that ROS levels elevated by Dox stress, decreased the PHD expression, which in turn enhances HIF1A-IGFBP3 signaling leading to downregulation of cellular survival and stimulation of apoptosis in cardiac cells.

**Figure 6:**
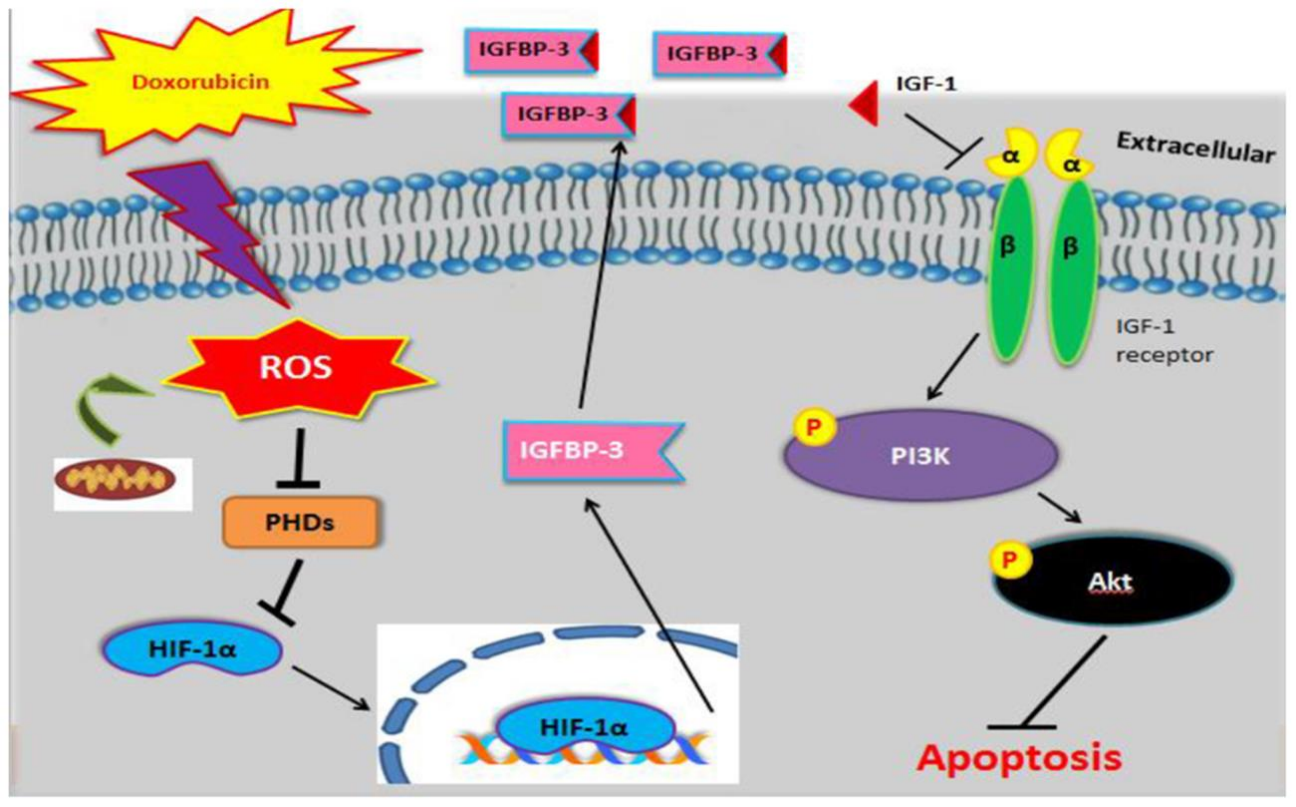
**Graphical representation illustrating the mechanism underlying Dox-induced cell apoptosis.** Dox induced ROS generation, which suppressed PHD. This stabilized nuclear HIF1A, promoted IGFBP3, and enhanced its extracellular association with IGF1. This interaction blocked survival signaling and resulted in cell apoptosis.

Altogether, the current study suggests that Dox-induced ROS generation inhibits PHD expression, leading to translocation of HIF1A to the nucleus and enhancing the expression as well as secretion of IGFBP3 and thereby blocks IGF1 pro-survival signaling, resulting in increased cardiac cell apoptosis (Figure 6).

## DISCUSSION

Numerous studies report that increased oxidative stress contributes to the Dox-induced cardiomyocyte apoptosis, which results in Dox-associated cardiomyopathy. Mitochondria represent the major documented source of ROS [24]. Dox induces mitochondrial ROS generation [25]. Additionally, mRNA and protein levels of IGFBP3 were upregulated by ROS production following Dox treatment. These findings indicate that ROS generation by Dox may stimulate IGFBP3 expression, resulting in cell apoptosis.

HIFs have been classified into three different categories: HIF1, HIF2, and HIF3. Among them, HIF1 is the primary oxygen-sensitive transcriptional activator [26]. The activity and stability of the HIF1A subunit are regulated by its post-translational modifications, such as ubiquitination, hydroxylation, acetylation, and phosphorylation. Free radicals, ROS, and/or reactive nitrogen species may upregulate HIF1A in tumors [27]. Under normoxic conditions, hydroxylation of two proline residues and acetylation of a lysine residue in the oxygen-dependent degradation domain of HIF1A trigger the association of HIF1A with pVHL E3 ligase complex, leading to HIF1A degradation via the ubiquitin- proteasome pathway [22]. Exposure to Dox stabilizes HIF1A and potentiates its interactions with co-activators, such as the cAMP response element-binding protein p300, affecting the expression of HIF1A target genes [28–32]. This interaction potentially explains why the expression levels of HIF1A and its target gene, IGFBP3, gradually increased following the exposure to Dox for 24 h in this study. Additionally, previous studies have suggested that other important mechanisms may also contribute to Dox-induced HIF1A accumulation in the normoxic conditions. The degradation of HIF1A depends on HIF-PH, which requires both Fe^2+^ and molecular oxygen [33, 34]. When Fe^2+^ is removed by chelating agents such as desferrioxamine or substituted with Co^2+^ or Ni^2+^, HIF1A degradation is inhibited. Therefore, desferrioxamine and CoCl_2_ mimic hypoxia effects. Dox is a strong iron chelator, therefore, the iron deficiency caused by is deficiency inhibits HIF-PH activity, causing normoxic HIF1A accumulation [35].

Some reports demonstrated that the increase of mitochondria ROS generation inhibited PHD expression, leading to HIF1A protein stabilization. Our findings are in agreement with these studies as Dox-induced mitochondrial ROS inhibited PHD expression, increasing HIF1A stability and thereby the downstream expression of IGFBP3. Apoptosis has been widely explored as a cause of cardiotoxicity by Dox [36], but the mechanisms whereby Dox downregulates IGF1 pathway are still unknown. IGF1 signaling pathway has been shown to exert protection from cell death. The IGF1/IGF1R signal transduction activates PI3K and AKT phosphorylation, thus enhancing cell survival. A previous study has demonstrated that IGFBP3 exerts anti-proliferative effects in several cell types by blocking the ability of IGF1 to activate IGF1R, a protein that stimulates cell proliferation [37]. In the present study, we investigated the involvement of the IGF1R/PI3K/AKT survival pathway downregulation in Dox-induced apoptosis. Our results support the hypothesis that Dox decreases the levels of phosphorylated IGF1R and AKT by activating HIF1A and IGFBP3 signaling. This hypothesis was particularly confirmed by the results of the silencing experiments, in which treatments with *Hif1a* or *Igfbp3* siRNA reversed the changes in IGF1R and AKT protein phosphorylation levels, inhibiting cardiac apoptosis. These observations strongly suggest that Dox induced increase in ROS generation suppresses myocardial survival pathway via HIF1A-IGFBP3-dependent signaling that enhances cell apoptosis. Our present data identify IGFBP3 as a key protein involved in Dox-induced cardiomyocyte apoptosis. In support of this view, we demonstrated that Dox increased the association of IGFBP3 with IGF1, whereas treatments with an anti-IGFBP3 antibody or *Igfbp3* siRNA reversed the downregulation of the IGF1 pro-survival signaling pathway by Dox resulting in oxidative stress and resistance to apoptosis. Inhibition of the IGF1 signaling pathway by Dox results in cardiotoxicity that manifests as heart failure, myocardial ischemia/infarction, and hypertension [38]. We showed that Dox-induced IGFBP3-dependent apoptosis was likely mediated by mitochondrial ROS generation and stabilization of HIF1A. Furthermore, decreasing IGFBP3 expression using the corresponding siRNA attenuated the expression of the pro-apoptotic proteins.

These findings suggest that Dox-induced cardiac apoptosis might be caused by the inhibition of the IGF1 survival pathway. Conclusively, the mechanisms regulating doxorubicin (Dox)-induced apoptosis in myocardial cells involve the downregulation of cardiac IGF1 pro-survival signaling, as a result of the increased association of extracellular IGF1 with IGFBP3, secreted at increased levels. Additionally, ROS-regulated PHD controls the intracellular activation of HIF1A-IGFBP3 signaling, IGFBP3 secretion, and cardiomyocyte apoptosis induced by Dox.

We believe that the current study findings help to understand the negative implications of Dox usage as an antibiotic as well as chemotherapeutic drug and thus encourage necessary efforts in cubing them. Our study underscore the role of protein, such as HIF1A as the mediator of Dox-induced apoptosis. This study provides information to develop potential therapeutic strategies to overcome the cardiotoxicity of Dox. However, future studies are required to explore such strategies and their effectiveness in other models and eventually in humans. Notable limitation of this study is that only control and Dox treated groups are used in the *in vivo* study. Besides, there is possible involvement of other signaling mechanisms in addition to ROS mediated HIF1A-IGFBP3 signaling axis. Therefore, further studies are required to validate the outcomes of the present study either using inhibitor models or employing knockdown/knockout model. In addition, it is vital to explore the other possible underlying mechanisms and their crosstalk with the HIF1A-IGFBP3 signaling involved in the cardiotoxicity caused by Dox.

## METHODS

### Cell culture experiments

H9c2, an rat embryo derived cardiomyoblast cell line acquired from the American Type Culture Collection (Manassas, VA, USA) were cultured in Dulbecco’s modified essential medium supplemented with 10% cosmic calf serum, 2 mM glutamine, 100 units/mL penicillin, and 100 μg/mL pyruvate in the humidified atmosphere of 95% air and 5% CO_2_ at 37°C. During the treatment, H9c2 cells were exposed to 1 μM Dox for 24 h, harvested, and extracted for further analysis.

### *In vivo* experiments

We used 8-week-old male Wistar rats that were purchased from the National Animal Breeding and Research Center (Taipei, Taiwan). All the animals were divided into two groups: normal group (*N* = 5) and Dox-treated group (*N* = 5). The animals were maintained under a 12-h light/dark cycle at an ambient temperature of 25°C. The treated group received intraperitoneal injections of Dox at a dose of 5 mg per kg body weight once a week for 6 weeks. The normal group received injections of the vehicle. All the experiments in this study were approved by the Ethical Committee Faculty of China Medical University [39, 40].

### Tissue extraction

Cardiac tissue samples from the control and Dox-challenged rats were homogenized for protein extraction in a lysis buffer at a concentration of 100 mg tissue/mL buffer. The homogenates were placed on ice for 15 min and then centrifuged at 12,000 × g for 30 min at 4°C. The supernatant was collected and stored at −80°C for further analysis.

### Lowry protein assay

Total protein concentration was determined with the Folin-Ciocalteu reagent for enhanced color development using the Lowry method. Diluted bovine serum albumin solutions (0, 0.1, 0.2, 0.3, 0.4, and 0.5 mg/mL) were used as standards. Proteins were first reacted with alkaline cupric sulfate in the presence of tartrate (2% Na-K tartrate: 1% CuSO_4_ × 5H_2_O: 2% Na_2_CO_3_ in 0.1 N NaOH = 1:1.98) for 10 min at room temperature. After the incubation, the Folin phenol reagent was added. The color enhancement occurs when the tetradentate copper complex transfers electrons to the phosphomolybdic/phosphotungstic acid complex. Following incubation for 40 min at room temperature in the dark, the absorbance was recorded at 750 nm.

### Immunohistochemistry (IHC)

IHC staining was performed as described previously [41, 42]. Cardiac tissues from the rats were deparaffinized with xylene, followed by rehydration using gradient series of alcohol. Thereafter, they were permeabilized, blocked, and washed twice with phosphate buffered saline (PBS) followed by primary antibody incubation for 1 h. Next, tissues were washed with PBS and incubated with horseradish peroxidase-conjugated avidin biotin complex using Vectastain Elite ABC Kit and NovaRED chromogen (Vector Laboratories, Burlingame, CA, USA) followed by hematoxylin staining to stain the nucleus. Finally, the photomicrographs were acquired and processed using microscopy (OLYMPUS^®^ BX53, Tokyo, Japan).

### Western blot analysis

Western blotting was performed as delineated in our previous studies [43, 44]. Briefly, cultured H9c2 cells with 70–90% confluency were harvested and lysed using a lysis buffer containing 50 mM Tris (pH 7.5), 0.5 M NaCl, 1.0 mM EDTA (pH 7.5), and 10% glycerol. The suspension was centrifuged at 12,000 rpm for 35 min and supernatant was collected. The collected proteins were separated by 10–12 % sodium dodecyl sulfate-polyacrylamide gel electrophoresis and transferred to a polyvinylidene difluoride (PVDF) membrane. Nonspecific protein binding was inhibited using 5% skimmed milk. The membrane was probed with specific primary antibodies in the blocking buffer at 4°C overnight. Thereafter, PVDF membranes were washed and incubated with the secondary antibody for 1 h followed by densitometric analysis using Fuji LAS 3000 imaging system. For repeated blotting, PVDF membranes were stripped with the Restore Western Blot Stripping Buffer at room temperature for 30 min.

### Transfection of plasmid and siRNAs

Cardiac cells with up to 50% confluency were replaced with fresh culture medium containing serum, treated with 1 μM Dox for 24 h, and transfected with either pCMV-PHD3 plasmid or siRNA against *Hif1a* and *Igfbp3,* using PureFection™ Nanotechnology-based Transfection Reagent (System Biosciences, CA, USA), according to the manufacturer’s protocol. In each experiment, the efficiency of gene overexpression was measured using western blot analysis. For the knockdown experiments, cells with 70–90% confluency were used Double-stranded siRNA sequences targeting *Hif1a* and *Igfbp3* mRNAs were obtained from EMD Millipore Corporation. The knockdown efficiency was confirmed using western blot analysis.

### Terminal deoxynucleotidyl transferase-mediated dUTP nick end labeling (TUNEL) assay

To assess apoptosis, H9c2 cells grown on 6 mm plates were fixed with 4% formaldehyde solution for 30 min at room temperature. After washing thrice with PBS, cells were permeabilized in 0.1% Triton X-100 solution in 0.1% sodium citrate for 10 min at 4°C. Following two washes with PBS, the samples were first incubated with the TUNEL for 1 h at 37°C in the dark. Then, cells were stained with DAPI for 2 min for nuclear detection. Fluorescence microscopy images of TUNEL-positive cardiac myocytes and apoptotic bodies were obtained using excitation wavelength in the range of 450–500 nm and recording the emission spectrum in the range of 515–565 nm (green).

### Measurement of mitochondrial ROS generation

Generation of mitochondrial ROS was evaluated in embryo-derived cardiac cells using MitoSOX (Invitrogen Molecular Probes, MA, USA). After H9c2 cells were challenged with Dox for 24 h, they were probed with MitoSOX reagent for 30 min at 37°C followed by DAPI staining for 5 min. ROS generation was evaluated using fluorescence microscopy (Olympus, Tokyo, Japan), by excitating the probes at 510 nm and recording the emission at 580 nm.

### Immunofluorescence

Immunofluorescence was executed to analyze the expression level of HIF1A. H9c2 cardiac cells cultured in chamber slides (SPL Life Sciences, Korea) were challenged with Dox for 24 h. They were then fixed in 4% paraformaldehyde for 1 h, permeabilized with 0.1% Triton X-100 for 5 min followed by blocking for 1 h to avoid nonspecific binding. Next, H9c2 cells were incubated with the HIF1α primary antibody for 1 h and Alexa Fluor 488 Red anti-rabbit IgG secondary antibody for 1 h. Lastly, cells were washed, and stained with DAPI, and then imaged to analyze the expression of HIF1A.

### Co-immunoprecipitation (Co-IP)

To evaluate protein-protein interactions, the total cellular extract from H9c2 cells was immunoprecipitation using Protein G magnetic beads (Millipore), according to the guidelines provided by the manufacturer. From the whole cell lysate 500 μg of protein was probed with 3 μg of the primary antibody overnight at 4°C. Finally, the protein complexes were washed with PBS and eluted at 95°C. The resultant elute was analyzed by the western blotting method as described above.

### Statistical analysis

Data values are expressed as the means ± standard deviation. The significance of the treatment effect was assessed by one-way analysis of variance using the GraphPad Prism5. All western blots and immunofluorescence images were analyzed using ImageJ software. Differences were considered to be statistically significant for *P* < 0.05.
